# Facile Fabrication of Titanium Carbide (Ti3C2)-Bismuth Vanadate (BiVO4) Nano-Coupled Oxides for Anti-cancer Activity

**DOI:** 10.7759/cureus.61492

**Published:** 2024-06-01

**Authors:** Nagubandi Lakshmi Anvitha, Geetha A, Vasugi S, Balachandran S, Ilangovar I.G.K

**Affiliations:** 1 Department of Physiology, Saveetha Dental College and Hospitals, Saveetha Institute of Medical and Technical Sciences (SIMATS) Saveetha University, Chennai, IND

**Keywords:** nano-coupled oxides, anti-cancer activity, energy dispersive spectrum, bismuth vanadate, ti3c2

## Abstract

Background

MXene is a newly discovered substance consisting of 2D transition metal carbides or nitrides, produced through the disintegration and etching of aluminum layers. It possesses numerous properties, including a high surface area, conductivity, strength, stiffness, negative zeta potential, and excellent volumetric capacitance. MXene is utilized in detecting anti-cancer medicine, while bismuth vanadate (BiVO_4_) is synthesized to form an optimized material for anti-cancer activity applications. BiVO_4_ exhibits visible light absorption, strong chemical stability, and non-toxic properties. However, when loaded onto target stem cells, it can cause skin and respiratory irritation.

Aim

This study aimed to evaluate the facile fabrication of titanium carbide (Ti_3_C_2_)-BiVO_4_ nanomaterials coupled with oxides for anti-cancer activity. Moreover, it aimed to create Ti_3_C_2_-BiVO_4_ nanomaterials in combination with oxides using X-ray diffraction (XRD) and scanning electron microscopy (SEM) to assess their potential as efficient and targeted anti-cancer agents.

Methods and materials

To prepare the 2D Ti_3_C_2_ MXene, 2.5 g of titanium aluminum carbide (Ti_3_AlC_2_) powder was dissolved in 60 mL of a 40% hydrofluoric acid (HF) solution in a polytetrafluoroethylene(PTFE) container. The etching process was made more efficient and completed in 24 hours by using a magnetic stirring system to keep the mixture stirred and heated continuously. The centrifugation was performed at 4000 rpm for five minutes. Subsequently, deionized water was used to wash the solution many times until its pH reached around 7. The appropriate Ti_3_C_2_ powder was made by vacuum drying the acquired sediment at 80°C for 24 hours. Monoclinic BiVO_4_ samples were synthesized via a hydrothermal method. Typically, 10 mmol of Bi(NO_3_)_3_.5H_2_O was dissolved in 100 mL of a 2 mol/L HNO_3_ solution and stirred uniformly. Subsequently, 10 mmol of ammonium metavanadate (NH_4_VO_3_) was added to the mixed solution. After being stirred for one hour, the mixture was transferred into a 100 mL sealed Teflon-lined stainless steel autoclave at 180°C for 16 hours. After cooling to room temperature, the sediment was washed three times with deionized water, ethanol, and acetone, respectively. Finally, the suspension was dried at 80°C, followed by calcination at 450°C for three hours to obtain BiVO_4_. Ti_3_C_2_-BiVO_4 _heterostructures were prepared by surface modification Ti_3_C_2 _using BiVO_4_ suspensions by a simple, cost-effective approach.

Results

Ti_3_C_2_ nanosheets were observed with BiVO_4_ particles, and the high crystalline nature of the compound was confirmed after XRD analysis and energy-dispersive spectroscopy (EDS) analysis. The compound was found to be pure without any impurities and exhibited anti-cancer activity.

Conclusion

The XRD, field emission scanning electron microscopy(FESEM), and EDS investigations provide an in-depth analysis of the structural, morphological, and compositional characteristics of Ti_3_C_2_-BiVO_4_ sheets. The XRD analysis proves the successful combination of different materials and the presence of crystalline phases. The FESEM imaging technique exposes the shape and arrangement of particles in sheets, while the EDS analysis verifies the elemental composition and uniform distribution. These investigations show that Ti_3_C_2_-BiVO_4_ composites have been successfully synthesized, indicating their potential for use in anti-cancer applications.

## Introduction

Cancer has a significant and extensive influence on human existence, impacting people, families, and society globally. Cancer is a leading cause of illness and death worldwide, with millions of new cases detected each year and millions of lives lost to the disease. Cancer may induce physical discomfort, exhaustion, and a range of other symptoms that vary based on the specific kind and stage of the illness [1]. Medical interventions such as chemotherapy, radiation treatment, and surgery may exert a considerable toll on the body and can result in substantial adverse reactions. Cancer may also lead to pain and discomfort, fatigue, cachexia, immune system suppression, and cognitive impairment that have an impact on the overall well-being and satisfaction of people [2]. Nevertheless, these obstacles in the fields of cancer research, prevention, early diagnosis, and therapy have resulted in enhancements in the rates of survival and the overall well-being of several individuals afflicted with cancer. Nevertheless, there is still a significant amount of work that has to be accomplished to tackle the worldwide impact of cancer and guarantee the fair and equal availability of cancer treatment for every person [3].

In 2024, the worldwide cancer burden will continue to be substantial. The World Health Organization (WHO) reported that there were an estimated 20 million new cancer diagnoses and 9.7 million deaths attributable to cancer in 2022. Approximately 53.5 million individuals worldwide were predicted to be living with cancer within five years after being diagnosed. Although surgery, chemotherapy, and radiotherapy are widely considered to be the most popular cancer treatment options, they are not always effective. Chemotherapy and radiotherapy, while very effective, have serious adverse effects when used [4]. The progressive resistance of cancer cells to treatment is one of the major issues with cancer treatment. The emergence of cancer cell lines resistant to chemotherapeutic drugs is a time-tested method for examining the causes of cytotoxicity and drug resistance in chemotherapeutic drugs. The quantity of healthy cells in a sample is known as cell viability, and the growth of cells is a key sign for comprehending the processes underlying the actions of certain genes, proteins, and pathways involved in a cell's ability to survive or die after being exposed to harmful substances. Chemotherapeutics' non-selective therapeutic strategy fundamentally makes it impossible to efficiently eradicate cancer stem cells (CSCs) [5].

If a chemotherapeutic agent is employed in combination therapy, the toxicity is greatly reduced because alternative routes will be targeted. In the end, this has a synergistic or cumulative effect, necessitating a lower therapeutic dosage of each medicine alone. Titanium carbide (Ti_3_C_2_) belongs to the group of two-dimensional materials called MXene. Although research on the anti-cancer properties of Ti_3_C_2_ is still in its nascent phase, several studies have shown its promising potential in this domain [6]. One suggested mechanism for the anti-cancer action of Ti_3_C_2_ is its capacity to trigger cancer cell apoptosis via many routes. Ti_3_C_2_ has shown cytotoxic properties against cancer cells by inducing apoptosis, a process of programmed cell death. In addition, Ti_3_C_2_ has the ability to trigger autophagy, which is a cellular mechanism that results in the breakdown and reuse of damaged or unneeded cellular parts, therefore promoting the death of cancer cells. Moreover, much research has been conducted on Ti_3_C_2_ nanoparticles to explore their potential as carriers for delivering drugs in the field of cancer treatment [7].

Ti_3_C_2_ nanoparticles possess distinctive physicochemical characteristics, such as a substantial surface area and functional groups, which enable them to effectively encapsulate and transport anti-cancer medicines to specific tumor locations. This strategy of tailored medication delivery decreases the negative effects on unintended areas and improves the effectiveness of anti-cancer treatments while decreasing their overall toxicity in the body. Furthermore, Ti_3_C_2_-based nanomaterials have been investigated for their capacities in photothermal therapy (PTT). Ti_3_C_2 _nanoparticles, when subjected to near-infrared (NIR) light, have the ability to produce heat, resulting in localized hyperthermia specifically inside tumors [8]. Hyperthermia caused by this method leads to the killing of cancer cells while preserving nearby healthy tissues, making PTT a very promising strategy for treating cancer. Ti_3_C_2_ has promise in its ability to combat cancer via many methods, such as direct cytotoxicity, medication delivery, and PTT. Bismuth vanadate (BiVO_4_) is an n-type semiconductor made up of elements that are comparatively common on Earth. Its advantages have led to its widespread use as a photoanode for photoelectrochemical (PEC) water splitting. It possesses a direct bandgap of 2.4 eV in the monoclinic phase, with a conduction band position near to 0 V vs. normal hydrogen electrode (NHE) (pH = 0) and a valence band position at +2.4 eV vs. NHE (pH = 0) [9]. BiVO_4_ is an adaptable material that has great potential for use in biomedical fields such as wound healing, photodynamic therapy, biosensing, and antibacterial treatment. Due to its unique characteristics, it is a promising material for future studies in photocatalysis and biocompatibility. Additional medical uses for BiVO_4_, including novel approaches to illness prevention and treatment, are anticipated as a result of ongoing developments in nanotechnology and materials science [10].

The high efficiency of bismuth (Bi) in treating pathogens, viruses, and cancerous tumors is a major factor in its use in medicine and healthcare. Compounds containing Bi have been widely shown to offer potential anti-cancer effects on malignant tumor cell lines. Bi (III) compounds' anti-cancer mechanism is primarily linked to the production of reactive oxygen species (ROS), a decrease in mitochondrial membrane potential, and the triggering of apoptosis. Bi-based anti-cancer drugs' precise mode of action, however, is still unknown [11]. Future research on Bi compounds as anti-cancer agents at the mechanistic and therapeutic levels may provide a new avenue. Although Bi is thought to be harmless, as previously indicated, prolonged exposure to it may have some adverse effects on human subjects. BiVO_4_ is a promising material because of its properties of visible light absorption, great chemical stability, non-toxicity, and low cost. We are going to synthesize Ti_3_C_2_ modified by BiVO_4_, and it will be characterized by different analytical methodologies. Optimized materials are used for anti-cancer activity in cancer cell lines. Ti_3_C_2_ electrochemical energy storage is ensured by its 2D form, excellent electrical characteristics, and biocompatibility [12].

The features of visible light absorption, strong chemical stability, non-toxicity, and inexpensive cost make BiVO_4_ a promising material. MXene is used in surface-enhanced Raman spectroscopy (SERS) to detect extremely small amounts of anti-cancer medicine. Favorable biocompatibility and ideal mechanical properties make them expand their application in the cancer therapy field. A material called MXene epigallocatechin gallate (EGCG) complex nanosheets may improve cancer PTT, and it has anti-inflammatory properties [13]. It was suggested that clinical cancer therapy and treatment are greatly aided by the quick, reliable detection of carcinogenic embryonic antigen, and it demonstrates that they have good bactericidal action, accelerating the healing of postoperative wounds without losing their ability to prevent recurrence. The current investigation comprised the preparation of Ti_3_C_2_-BiVO_4_ heterostructures utilizing a modified synthesis process that involved hydrofluoric acid (HF) etching followed by a hydrothermal technique [14]. The Ti_3_C_2_-BiVO_4_ nanosheets have been prepared and have exceptional behavior in both synthetic procedures and characterization studies. The presence of Ti_3_C_2_-BiVO_4_ nanomaterials was verified using X-ray diffraction (XRD), scanning electron microscopy (SEM), and energy-dispersive spectroscopy (EDS) investigation, which included examining their crystalline behavior, phase, surface morphology, and elemental composition. The study examined the anti-cancer characteristics of Ti_3_C_2_-BiVO_4_ that was artificially produced. A cell viability study was conducted in vitro on colorectal cancer (CRC) cell lines to assess the biocompatibility of Ti_3_C_2_-BiVO_4_.

## Materials and methods

The preparation of Ti_3_C_2_ using HF is to etch the layers of aluminum (Al) from titanium aluminum carbide (Ti_3_AlC_2_) (MAX phases), where "M" stands for a transition metal (titanium (Ti)) and "A" for an element (Al). To prepare the 2D Ti_3_C_2_ MXene, 2.5 g of Ti_3_AlC_2_ powder was dissolved in 60 mL of a 40% HF solution in a polytetrafluoroethylene (PTFE) container. The etching process was made more efficient and completed in 24 hours by using a magnetic stirring system to keep the mixture stirred and heated continuously. The centrifugation was performed at 4000 rpm for five minutes. Subsequently, deionized water was used to wash the solution many times until its pH reached around 7. The appropriate Ti_3_C_2_ powder was made by vacuum drying the acquired sediment at 80°C for 24 hours.

Monoclinic BiVO_4_ materials were synthesized via a hydrothermal method. Typically, 10 mmol of bismuth nitrate (Bi(NO_3_)_3_.5H_2_O) was dissolved in 100 mL of a 2 mol/L HNO_3_ solution and stirred uniformly. Subsequently, 10 mmol of ammonium metavanadate (NH_4_VO_3_) was added to the mixed solution. After being stirred for one hour, the mixture was transferred into a 100 mL sealed Teflon-lined stainless steel autoclave at 180°C for 16 hours. After cooling to room temperature, the precipitate was washed three times with deionized water, ethanol, and acetone, respectively (solution A). Finally, the precipitate was dried at 80°C, followed by calcination at 450°C for three hours to obtain BiVO_4_.

The prepared Ti_3_C_2_ was dispersed in 20 mL of dimethyl sulfoxide (DMSO) and sonicated for three hours (solution B). Then the solution B was dropwise added to the solution A and stirred for two hours at 90°C. The reaction mixture is transferred to a hydrothermal process at 180°C for 12 hours. After cooling to room temperature, the precipitate was washed three times with deionized water, ethanol, and acetone. After preparation, the materials are characterized using XRD, SEM, and EDS analysis. The XRD study allows us to explain the crystalline behavior and phase purity of the material. SEM images are utilized to examine the surface morphology and evaluate the average particle size, as well as to determine the pressure exerted on the surface. EDS analysis confirmed the elements' composition.

The 3-(4,5-dimethylthiazol-2-yl)-2,5-diphenyltetrazolium bromide (MTT) test is often used to check for cell viability and proliferation. It uses colorimetric assays to measure how metabolically active cells change a yellow tetrazolium salt called MTT into formazan crystals. The CRC cells (HCT116) are evenly distributed over the wells of a multi-well plate and left to adhere for the night. Different concentrations of Ti_3_C_2_-BiVO_4_ nanosheets are used to treat cells, usually ranging from very low to very high. To compare the results, we also include control groups of cells that were either not treated or treated with vehicle control, such as culture media. It is common practice to incubate cells with nanosheets for a certain amount of time after treatment, usually 48 hours, so that the nanosheets can exert their cytotoxic effects. The MTT reagent is added to each well after the incubation time, and the cells are incubated for an additional duration, typically 2-4 hours. Purple formazan crystals are formed when metabolically active cells decrease the MTT reagent. An aspiration of the culture media containing the MTT reagent is performed after incubation. A solvent, such as DMSO, is then added to dissolve the formazan crystals, producing a solution that is purple in color. Using a microplate reader, the absorbance of the purple formazan solution is measured spectrophotometrically at a wavelength usually about 570 nm. Optimal cell density in a culture is a good indicator of absorbance intensity. We can determine the relative percentages of cell viability or cytotoxicity from the MTT absorbance readings compared to the control groups. In order to find the concentration of Ti_3_C_2_-BiVO_4_ nanosheets that inhibits cell viability by half, a dose-response curve is constructed, and the IC50 value is calculated.

## Results

XRD analysis

**Figure 1:**
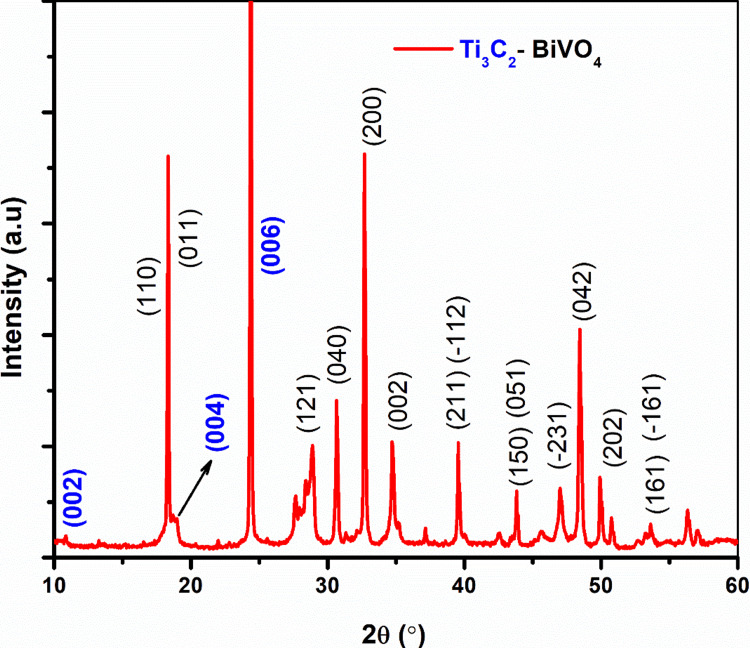
XRD pattern of Ti3C2-BiVO4 XRD: X-ray diffraction; BiVO_4_: bismuth vanadate; Ti_3_C_2_-BiVO_4_: titanium carbide (MXene)-bismuth vanadate

XRD analysis determined the crystalline behavior of the prepared Ti_3_C_2_-BiVO_4_ materials. Figure 1 shows that the most prominent diffraction peak for the (104) plane of Ti_3_AlC_2_, 2ϴ at 39.0°, disappeared after HF treatment, suggesting the elimination of the Al layers of MAX phase Ti_3_AlC_2_ [15]. The diffraction peaks at 19.1°, corresponding to the (004) plane, exhibited a downward shift in their angles and increased broadness. This phenomenon was attributed to an expansion in the distance between layers of Ti_3_C_2_ [16].

The peaks associated with BiVO_4_ were seen at 2θ = 18.3°, 18.5°, 28.79°, 31.7°, 32.6°, 34.48°, 36.19°, 40.1°, 43.54°, 47.1°, 48.6°, 50.6°, 55.4°, and 58.85°, which correspond to the (110), (011), (121), (040), (200), (002), (211), (-112), (150), (051), (-231), (042), (202), (161), and (-161) diffraction planes, respectively. The diffraction pattern is in good agreement with Joint Committee on Powder Diffraction Standards (JCPDS) card no. 14-0688, which confirms the presence of the monoclinic phase of BiVO_4_. The sharp peaks indicate the high crystalline nature of Ti_3_C_2_-BiVO_4_. The intense and distinct peak patterns closely correspond to a monoclinic phase of BiVO_4_. The diffraction planes of (002), (004), and (006) confirm the formation of Ti_3_C_2_. No other impurities are present in the Ti_3_C_2_-BiVO_4_ materials (Figure 1).

EDS analysis

**Figure 2:**
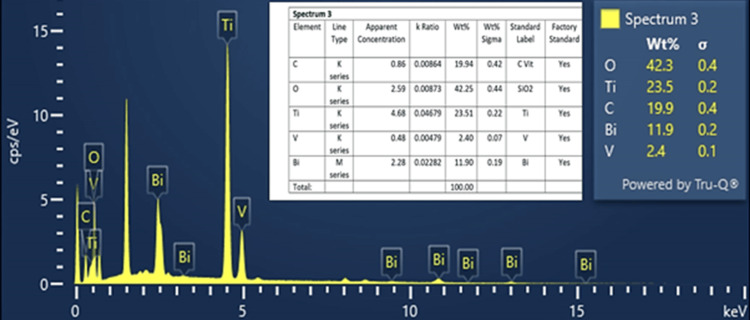
EDS analysis of Ti3C2 and BiVO4 EDS: energy-dispersive spectroscopy, Ti_3_C_2_: titanium carbide; BiVO_4_: bismuth vanadate; O: oxygen; Ti: titanium; C: carbon; Bi: bismuth; V: vanadium

EDS analysis identifies and quantifies the elements present in the Ti_3_C_2_-BiVO_4_ sample. The primary constituents of Ti_3_C_2_ are Ti and carbon (C). The EDS spectrum displays characteristic X-ray peaks corresponding to these elements. In this spectrum, Ti was found to be 23.51%, C 19.94%, oxygen (O) 42.25%, Bi 11.9%, and vanadium (V) 2.04%. All these compounds are depicted in the below EDS analysis. This confirms the presence of only the elements of the compound, indicating no impurities were observed (Figure 2).

SEM analysis

**Figure 3:**
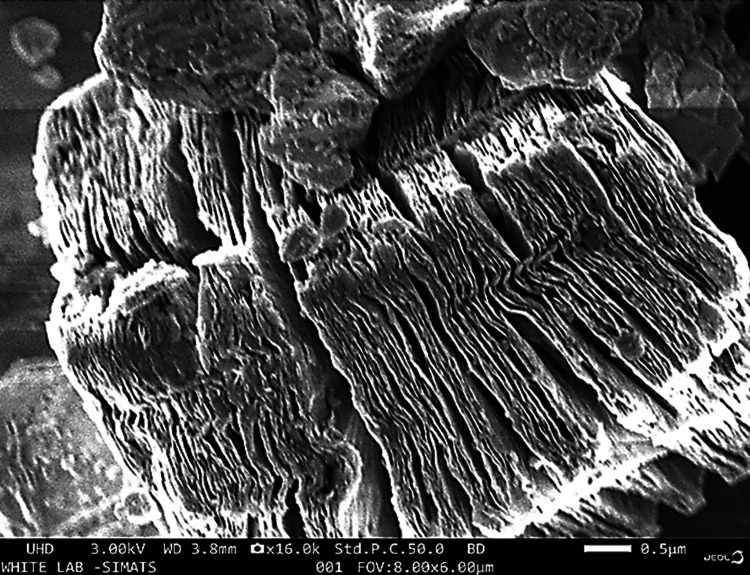
SEM images of the Ti3C2-BiVO4 SEM: scanning electron microscopy; Ti_3_C_2_-BiVO_4_: titanium carbide (MXene)-bismuth vanadate

Ti_3_C_2_-BiVO_4_ materials typically exhibit a sheet-like structure with a large lateral size. SEM images can capture the presence of interconnected networks of nanosheets, providing insights into the exfoliation and delamination processes during Ti_3_C_2_-MXene synthesis. It was analyzed and found to be 0.5 micrometers thick. Many voids and gaps were observed in the sheets. BiVO_4_ particles can be seen on and between the Ti_3_C_2_ sheets. The SEM images of Ti_3_C_2_-BiVO_4_ composites reveal a sheet-like structure that is composed of interconnecting layers or flakes with varied thicknesses. The Ti_3_C_2_ layers are a framework on which BiVO_4_ nanoparticles or nanosheets are attached. This configuration has several benefits for anti-cancer purposes. The sheet-like morphology of Ti_3_C_2_-BiVO_4_ composites offers a large surface area, which is advantageous for enhancing the quantity of active sites accessible for anti-cancer applications. The combination of Ti_3_C_2_ and BiVO_4_ may lead to synergistic effects, where the distinct features of each component complement and increase the total efficiency (Figure 3).

Anti-cancer activity

**Figure 4:**
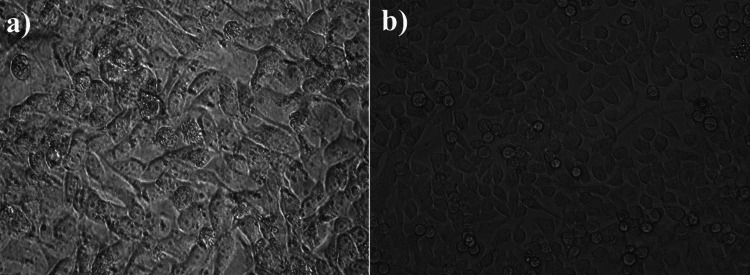
Anti-cancer activity of the Ti3C2-BiVO4 (a) control and (b) after 48 hours BiVO_4_: bismuth vanadate; Ti_3_C_2_-BiVO_4_: titanium carbide (MXene)-bismuth vanadate

It depicts cell death, demonstrating that this compound exhibits good anti-cancer activity when detected with the MTT assay using colon cancer cells (Figure 4). The unique physicochemical features and capacity to selectively target cancer cells of nanomaterials, including Ti_3_C_2_-BiVO_4_ nanosheets, have made them a viable method for cancer treatment. In this article, we go into the specifics of how Ti_3_C_2_-BiVO_4_ nanosheets fight CRC cells. The photocatalytic characteristics of Ti_3_C_2_-BiVO_4_ nanosheets allow them to produce ROS when exposed to light. DNA damage, lipid peroxidation, and protein oxidation are outcomes of ROS induction in cancer cells, which includes singlet oxygen (~1O_2_) and superoxide radicals (O_2_^-^). Cancer cells undergo apoptotic cell death when this oxidative stress upsets their balance. Cancer cells are able to absorb Ti_3_C_2_-BiVO_4_ nanosheets because of their small size and distinctive physicochemical characteristics. Nanosheets have anti-cancer properties when they are absorbed and localized to the cytoplasm and nucleus. To ensure the effective delivery of therapeutic payloads to target CRC cells, the addition of BiVO_4_ increases the nanosheets' biocompatibility and stability. By causing mitochondrial membrane depolarization, reducing adenosine triphosphate (ATP) generation, and increasing cytochrome c release into the cytoplasm, Ti_3_C_2_-BiVO_4_ nanosheets interfere with mitochondrial activity in CRC cells. Cell death, DNA breakage, and caspase activation are the outcomes of these processes, which trigger the intrinsic apoptotic pathway. G0/G1 or G2/M cell cycle arrest is induced in CRC cells by treatment with Ti_3_C_2_-BiVO_4_ nanosheets. Cell cycle inhibitors like p21 and p27 are upregulated, whereas cyclins and cyclin-dependent kinases (CDKs) are downregulated, mediating this arrest. Consequently, CRC cells are rendered incapable of undergoing cell cycle progression and apoptotic cell death. Key signaling pathways involved in the advancement of CRC, such as the PI3K/AKT/mTOR, MAPK/ERK, and Wnt/β-catenin pathways, are modulated by Ti_3_C_2_-BiVO_4_ nanosheets. The nanosheets have strong anti-cancer properties by reducing CRC cell motility, invasion, and survival via blocking the activation of these pathways. Ti_3_C_2_-BiVO_4_ nanosheets improve the immune response to cancer in CRC, demonstrating immunomodulatory characteristics. The immune system is able to identify and eliminate CRC cells because the nanosheets activate and infiltrate cytotoxic T lymphocytes (CTLs), natural killer (NK) cells, and dendritic cells (DCs) into the tumor microenvironment. Against CRC cells, Ti_3_C_2_-BiVO_4_ nanosheets induce ROS-mediated oxidative stress, alter mitochondrial function, stop the cell cycle, block signaling pathways, and modulate the immunological response, among other multi-faceted anti-cancer activities. These results emphasize the promise of Ti_3_C_2_-BiVO_4_ nanosheets as an innovative approach to treating CRC.

## Discussion

Jastrzębska et al. analyzed the cancer applications of MXene and found that MXene has great anti-cancer potential and promising in vitro and in vivo results, which help in the precise drug delivery of MXene [17]. Badrigilan et al. analyzed the role of MXene in breast cancer and found that it reduced cytotoxicity and showed a great surface-to-volume ratio [18]. Zhang et al. analyzed the anti-cancer properties of MXene which showed that MXene kills human cancer cells by using certain mechanisms [19]. Jastrzębska et al. analyzed the cytotoxicity of delaminated MXene and obtained that MXene used the oxidative stress method to kill the cancerous cells present and reduce the cytotoxicity to an extent [17]. Jolly et al. reviewed the advances in cancer treatment using Bi-based nanoparticles and captured that, due to their low toxicity, X-ray sensitivity, high atomic number, near-infrared-driven semiconductor qualities, and low cost, multifunctional nanomaterials based on Bi have significant potential for the treatment and diagnostics of cancer [20]. Here, a thorough analysis of recent developments in the medical uses of Bi-based nanomaterials is presented, covering such topics as assessments of in-tumor site accumulation, tumor targeting, and therapeutic performance, as well as the features, advantages, and drawbacks of major monotherapies mediated by Bi-based nanomaterial. Badrigilan et al. reported that an efficient theranostic for clinical cancer treatment can be obtained by combining high-performance CT imaging and PTT into one nanoprobe [18]. Such nanotheranostics have acceptable blood compatibility, cytotoxicity, and physiological dispersity. Bi nanoparticles have a strong and constant absorbance profile in the NIR region, high photostability, and remarkable photothermal efficiency of 30.0%. The outcomes show graphene quantum dots (GQDs)-Bi nanoparticles' promise as a successful therapeutic nanoagent for CT imaging and cancer PTT [21]. Shumin after analyzing reported that due to their favorable characteristics, including low toxicity, excellent X-ray absorption, and ease of fabrication, Bi-based nanoparticles are extensively researched in the detection and therapy of cancer. However, due to poor tumor site targeting, lengthy retention-induced systemic toxicity, and immune resistance, pure Bi alone cannot produce both effective and safe radiotherapy results. When paired with various therapies like PTT and high-intensity focused ultrasound (HIFU), bio-based nanoparticles show synergistic anti-cancer potential. Zhang reported regarding XRD that the peaks of high-purity Ti_3_AlC_2_ powders can be seen with only a small amount of TiC and the XRD patterns reflect the level of Al etching. After 40 hours of HF reaction, the peak at 239° that corresponds to Ti_3_AlC_2_'s distinctive peak (104) completely vanished, indicating that Al has been removed from the compound. Parallel to this, the (002) diffraction peaks at 29.5° [22]. Jolly et al. reported that the XRD pattern showed that Al_2_O_3_, TiC, and Ti_3_AIC_2_ were present. Following the trend shown by earlier work producing Ti_3_AIC_2_ and Ti_2_AIC from TiO_2_, the XRD patterns from the 3.0:5.5:1.9 and 3.0:6.0:2.0 ratios and the previously mentioned temperature changes showed more Al_2_O_3_ and TiC relative to Ti_3_AIC_2_ [20]. Evident from the lower intensity of the TiC diffraction peaks (around 2ϴ of 36.0°, 41.9°, 60.7°, and 72.6°) compared to those detected for the 3.0:5.5:1.9 ratio, a lower content of TiC was achieved for 3.0:6.0:1.9 [23].

Limitation

Enhancing the yield, repeatability, and scalability of the fabrication process through the optimization of the synthesis method; improving the stability, biocompatibility, and targeting abilities of Ti_3_C_2_-BiVO_4_ nanomaterial-linked oxides by investigating surface modification strategies; and exploring potential synergies by integrating Ti_3_C_2_-BiVO_4_ nanomaterials with other therapeutic methods like radiation therapy, immunotherapy, or chemotherapy are the efforts that could contribute to the development of more effective and versatile cancer treatment strategies in the future.

## Conclusions

The investigation demonstrated the simple synthesis of Ti_3_C_2_-BiVO₄ and their potential for use in cancer treatment. A composite material with improved anti-cancer capabilities was produced by integrating Ti_3_C_2_ MXenes with BiVO_4_ using an efficient and easy synthesis technique. The fabrication of the Ti_3_C_2_-BiVO₄ composite was confirmed by the XRD study. The final product was determined to include both Ti_3_C_2_ and BiVO₄ because characteristic peaks corresponding to both components were detected. In addition to confirming the high purity of Ti_3_C_2_-BiVO_4_ materials, the XRD patterns showed no obvious impurities. The long-term performance of the composite under evaluation circumstances was suggested by the well-maintained crystalline structure and phase composition. Using SEM, the morphology and structural properties of the Ti_3_C_2_-BiVO₄ were thoroughly examined. The SEM images showed that the Ti_3_C_2_ sheets had a uniform distribution of BiVO₄ nanoparticles, creating a composite that was well-dispersed. By increasing the surface area and offering active sites, the morphology study showed that the BiVO₄ nanoparticles were evenly anchored on the Ti_3_C_2_ surface. In order to enhance the material's interaction with cancer cells during photodynamic treatment, this morphological trait is essential. The Ti_3_C_2_-BiVO₄ composite's elemental composition was validated using EDS studies. By displaying clear peaks for Ti, C, Bi, V, and O in the EDS spectra, the Ti_3_C_2_ matrix was shown to have successfully incorporated BiVO₄. The composite caused cancer cells to undergo oxidative stress and cell death by producing ROS. Ti_3_C_2_ and BiVO₄ worked together to increase the composite's photodynamic treatment efficacy by boosting ROS generation efficiency. This composite shows promise for clinical use in cancer therapy due to its biocompatibility and low toxicity to healthy cells.
